# Healthful Plant-Based Diets and Cognitive Function in Older Adults: Mediation by Nutritional Status and Modification by Urban–Suburban Location and Gender in a Shanghai Community-Based Study

**DOI:** 10.3390/nu18020316

**Published:** 2026-01-19

**Authors:** Zishuo Huang, Gonghang Qiu, Borui Yang, Ye Shao, Shuna Lin, Huimin Zhou, Liang Sun, Ying Wang

**Affiliations:** 1 School of Public Health, Fudan University, Shanghai 200030, China; rowanh990207@163.com (Z.H.); q18807039240@126.com (G.Q.); 24211020249@m.fudan.edu.cn (B.Y.); 23211020179@m.fudan.edu.cn (Y.S.); 23211020244@m.fudan.edu.cn (S.L.); 2 Key Laboratory of Public Health Safety, NHC Key Laboratory of Health Technology Assessment, Shanghai 200030, China; 3 Key Laboratory of Environmental Pollution Monitoring and Disease Control, School of Public Health, Ministry of Education, Guizhou Medical University, Guiyang 561113, China; zhouhuimin0814@163.com; 4 The NHC Key Laboratory of Geriatrics, Institute of Geriatric Medicine, Chinese Academy of Medical Sciences, Beijing Hospital/National Center of Gerontology of National Health Commission, Beijing 100730, China

**Keywords:** healthful plant-based diet index, cognitive function, older adults, nutritional status, gender differences, urban–suburban differences

## Abstract

Background and aims: Amid global aging, the role of diet in cognitive health is crucial. The healthful plant-based diet index (hPDI) is linked to cardiometabolic benefits, but its association with cognitive function in older adults, particularly through nutritional status and across different socio-geographic contexts, remains unclear. This study investigated the association between hPDI and multidimensional cognitive function, the mediating role of nutritional status, and potential associated modifications by urban–suburban location and gender. Methods: A community-based cross-sectional study was conducted in Shanghai, China, involving 2079 older adults (aged ≥60). Dietary intake was assessed by a validated food frequency questionnaire (FFQ) to calculate hPDI. Cognitive function was evaluated using the Mini-Mental State Examination (MMSE), Montreal Cognitive Assessment-Basic (MoCA-B), and Clinical Dementia Rating (CDR). Nutritional status was measured by the Mini Nutritional Assessment (MNA). Hierarchical regression, interaction, and mediation analyses were performed, adjusting for comprehensive covariates based on social determinants of health (SDoH). Results: Higher hPDI was significantly associated with better cognitive scores (MMSE: β = 0.083, *p* < 0.001; MoCA-B: β = 0.069, *p* < 0.001) and lower odds of worse CDR (OR = 0.944, *p* < 0.001) in fully adjusted models. In the cross-sectional mediation analysis, MNA statistically mediated a significant proportion of the observed associations (MMSE: 41.25%; MoCA-B: 53.68%; CDR: 38.98%). The protective association was consistent across urban and suburban areas. However, a significant three-way interaction (hPDI × Gender × Area, *p* < 0.01) was found, with no cognitive benefit observed for males in suburban areas. Conclusions: Adherence to a healthful plant-based diet is associated with better cognitive function in older adults, partly statistically mediated by improved nutritional status. While this association is geographically equitable in Shanghai, suburban males do not appear to benefit, highlighting the need for gender- and context-sensitive dietary interventions.

## 1. Background

### 1.1. Plant-Based Diet Quality and Cognitive Health in Aging Populations: Inconsistent Evidence and Underexplored Pathways

Against the backdrop of global population aging, Alzheimer’s disease (AD)—accounting for 60–70% of dementia cases—imposes a severe and growing public health and socioeconomic burden [1,2,3]. This reality underscores the critical need to target modifiable risk factors, given that up to 45% of cognitive impairment may be preventable through early non-pharmaceutical intervention [4]. Among these factors, dietary patterns represent a promising target due to their broad accessibility, with plant-based diets attracting particular interest [5]. To address the limitations of overly generalized definitions, the healthful plant-based diet index (hPDI) has been developed to systematically differentiate between healthful plant foods (e.g., whole grains, vegetables, fruits, legumes, nuts) and unhealthful plant-based or animal-based items [6,7]. While hPDI is well-established in cardiometabolic research—linked, for example, to a 34–45% reduction in type 2 diabetes risk—its relationship with cognitive function remains inconsistent and controversial across studies [8]. A 2025 systematic review and meta-analysis of four cohort studies on strict plant-based diets and cognitive outcomes found inconsistent results, with three studies reporting statistically significant protective associations, while the remainder showed no significant link [7]. These inconsistencies suggested that the association between diet and brain health may not be explained by a direct pathway but rather multiple statistically correlated mechanisms across nutritional dimensions, and that it further interacts with factors such as education, socioeconomic status, and mental health [9]. The full complexity of these relationships remains to be clarified.

### 1.2. Social Determinants of Health as Key Confounders in Diet–Cognition Association

Dietary behavior is deeply embedded in and systematically shaped by the social, economic, and environmental contexts captured by social determinants of health (SDoH) [10]. Factors such as sociodemographic characteristics, economic status, health-related behaviors, and psychological conditions are established predictors of cognitive function and simultaneously shape food access, dietary preferences, and long-term eating patterns. In research on plant-based diets and cognitive health, failing to adequately account for these multidimensional confounders may introduce residual confounding, obscuring the true association [11]. Operationalizing the broad SDoH, economic status, and environmental contexts into a composite measure for use as a statistical covariate is methodologically and scientifically crucial. It allows researchers to isolate external confounders and thereby clarify the independent protective association between the hPDI index and cognitive health.

### 1.3. Nutritional Status as a Potential Mediator Between Diet and Cognitive Health

Beyond controlling for confounding factors, understanding how the hPDI relates to cognitive health is essential, with overall nutritional status posited as a key intermediary. The Mini Nutritional Assessment (MNA) is a particularly suitable measure for this mediating role, as it is a validated, multidimensional tool specifically designed for older adults that integrates anthropometric, dietary, and health-related indicators into a composite score reflecting overall nutritional risk or adequacy [12,13]. Theoretically, a high-hPDI dietary pattern—rich in whole grains, vegetables, fruits, legumes, and nuts—was associated with improved nutrient intake and dietary quality, which should be captured by a higher MNA score [12,13]. Adherence to such a diet is linked to better nutrient intake and lower malnutrition risk [14]. Notably, MNA scores are lower in individuals with AD compared to cognitively normal peers and decline further with disease progression, suggesting its relevance not only as a nutritional assessment tool but also as a marker linked to cognitive trajectories [15]. Examining the pathway from hPDI to nutritional status (MNA) to cognitive outcomes could thus help clarify one potential route through which dietary patterns correlate with cognitive health and inform practical dietary strategies for older populations.

### 1.4. Exploring Geographic and Gender Dimensions in Diet–Cognition Associations Using Multidimensional Assessment

Drawing upon the SDoH model and theories of health inequality, the potential benefits derived from diet are not uniformly distributed but are influenced by socioeconomic status, resource access, and sociocultural norms such as gender roles [16]. In megacities like Shanghai, gradients in resource distribution and lifestyle patterns exist between urban and suburban areas, and traditional gender-role beliefs may further shape dietary behaviors differentially across regions [17]. Against this backdrop, a core question that remains systematically underexplored emerges: does the association between an hPDI and cognitive function—potentially mediated by nutritional status—vary by geographical location and gender? To address this and comprehensively capture diet-related associations, this study employs a multidimensional cognitive assessment strategy, utilizing the Mini-Mental State Examination (MMSE) for global screening [18], the Montreal Cognitive Assessment-Basic for executive function (MoCA-B) [19], and the Clinical Dementia Rating (CDR) for clinical staging [20], thereby mitigating the limitations of any single instrument.

### 1.5. Study Aims and Multilevel Hypotheses

To address these research gaps, this study will conduct a multilevel analysis using a community-based sample of older adults from central urban, inner suburban, and outer suburban areas of Shanghai. The specific objectives are: (1) to examine the association between hPDI and multidimensional cognitive function (assessed via MMSE, MoCA-B, and CDR); (2) to explore whether nutritional status (MNA) statistically mediates this association; (3) to evaluate the stability of this mediation model across urban–suburban regions; and (4) as a core innovative aim, to investigate potential gender-specific differences in this pathway, with particular attention to heterogeneous patterns in suburban settings. By elucidating how associations between hPDI and cognitive outcomes may vary across socio-geographical and gender subgroups, this research aims to inform the development of targeted, equitable nutritional strategies for cognitive health in aging urban populations.

## 2. Materials and Methods

### 2.1. Study Samples

Using a multistage cluster random sampling method, the study recruited community-dwelling older adults [21]. The investigation was conducted in 2025. The sampling procedure comprised three stages. First, Shanghai was stratified into three stratums based on urbanization level: city center, inner suburbs, and outer suburbs, with two subdistricts randomly selected from each tier. Second, within each selected subdistrict, two communities were randomly chosen as investigation sites. Third, within each sampled community, a roster of all residents aged 60 years or older was compiled, stratified by age group and gender. Participants were then randomly selected from each stratum proportional to its size. All data were collected via face-to-face, household-administered questionnaires. The sample encompassed older adults across a cognitive spectrum ranging from normal cognition to mild dementia. While this inclusive sampling strategy enhances the generalizability of findings to the broader aging population, it also recognizes that cognitive status may shape dietary behaviors—a point addressed analytically and further discussed in the limitations section.

The sample size calculation was conducted for a prevalence study using the formula: $N=\frac{\mu_{\alpha }^{2}\times P(1-P)}{\delta^{2}}\times deff$. We used the national prevalence (p) of MCI and dementia in Chinese older adults, which is 21.69% [22]. A 95% confidence level (Z = 1.96) and a relative error of 15% (resulting in an absolute error, δ = 0.032535) were applied. Based on the high within-community homogeneity of the older population regarding key study variables and the use of stratified random sampling to minimize between-cluster variance, a design effect (deff) of 1 was assumed, which determined that a minimum of 614 participants were needed per sampling stratum. The sampling framework was stratified by geographic area into three layers: city center, inner suburbs, and outer suburbs. Accounting for a projected non-response and invalid questionnaire rate of 10%, the total sample size was calculated as follows: (614 participants/stratum × 3 strata)/(1 − 0.10) ≈ 2055 participants.

To maintain a near 1:1:1 sampling ratio across three geographic strata, six subdistricts were selected (two per stratum). Within each subdistrict, two communities were randomly chosen. Approximately 168 older adults were randomly sampled from each of the twelve communities, reflecting local age and gender distributions. The final analytical sample included 2096 participants, with 2079 providing completed questionnaires. Detailed sampling locations are presented in the Supplementary Table S1.

### 2.2. Measurement

#### 2.2.1. Dependent Variable: Plant-Based Diet Index

Dietary intake was assessed using a validated, simplified food frequency questionnaire (FFQ). Based on established health effects, 20 food groups from the FFQ were categorized into three classes: healthful plant foods encompass whole grains, fruits, vegetables, nuts, legumes, healthy vegetable oils, and tomatoes, all in their minimally processed forms, as well as unsweetened tea and black coffee are included in this category; unhealthful plant foods, characterized by high processing, added sugars, refined carbohydrates, or unhealthy fats, include fruit juices (even 100% juice, due to low fiber and high glycemic load), sugar-sweetened beverages, refined grains, highly processed potato products, and sweets or desserts; Animal foods included animal fats (e.g., butter, lard), milk and dairy products (such as cream and ice cream), eggs, fish and seafood, meat, as well as other animal-based foods like pizza, which typically contains cheese and/or meat. The intake frequency for each food group was quantified using a 5-point scale, where “almost every day” was assigned a score of 5, “≥1 time/week” a score of 4, “≥1 time/month” a score of 3, “occasionally” a score of 2, and “rarely or never” a score of 1 [23].

The overall hPDI was derived by summing scores across all food groups according to the following scoring principle: positive scores (ranging from 1 to 5 as described above) were assigned for healthful plant food groups, whereas reverse scoring (i.e., 1 = 5, 2 = 4, 3 = 3, 4 = 2, 5 = 1) was applied to both unhealthful plant food groups and animal food groups. The final hPDI score was calculated as the sum of these adjusted scores, with higher values indicating stronger adherence to a healthful plant-based dietary pattern. In the present study, hPDI was used as the primary dietary exposure [24].

#### 2.2.2. Independent Variable: Cognitive Function

Cognitive function was assessed using three validated instruments, each selected to capture complementary dimensions of cognition and aligned with standard practices in nutritional epidemiology.

The MMSE was administered as a broad screening tool for global cognitive health. This 30-point instrument evaluates several basic domains: orientation to time and place, immediate and short-term recall, attention and calculation (e.g., serial subtraction), language abilities (including naming, repetition, reading, and writing), and visuospatial construction [18].

For a more detailed profile of specific cognitive domains—particularly executive function, attention, and higher-order processing—the MoCA-B was used. Also scored out of 30 points, the MoCA-B includes tasks assessing executive functions, visuospatial skills, naming, memory (with a delayed recall component), sustained attention, language, abstraction, and orientation. Its design is adapted for populations with limited education, enhancing its suitability in community-based studies of older adults [19].

Finally, disease staging and functional impact were evaluated using the CDR scale, a semi-structured interview that quantifies severity of cognitive and functional impairment. The global CDR score is derived from six domains—memory, orientation, judgment and problem solving, community affairs, home and hobbies, and personal care—and classifies participants as follows: 0 (no dementia), 0.5 (very mild), 1 (mild), 2 (moderate), or 3 (severe dementia) [20].

#### 2.2.3. Mediating Variable: Assessment of Nutritional Status

Nutritional status was assessed using the full MNA, a validated and widely used screening instrument developed specifically for older adults. The tool consists of 18 items organized into four domains [25]: (1) anthropometry (body mass index, mid-arm and calf circumferences, and recent weight loss); (2) general health status (lifestyle, medication use, mobility, and psychological stress); (3) dietary habits (number of meals, food and fluid intake, and eating autonomy); and (4) self-perceived health and nutritional status. Total scores range from 0 to 30, with higher scores indicating better nutritional status.

In this study, the MNA was positioned as a mediator reflecting comprehensive nutritional status, rather than merely a downstream correlate of dietary quality. This approach is justified by the distinct constructs underlying the hPDI and the MNA: while the hPDI specifically quantifies plant-based dietary patterns, focusing exclusively on the intake of plant-based food groups [23]. The MNA is a multidimensional tool assessing global nutritional risk. Its composite score integrates not only brief dietary items but also key physiological and psychosocial indicators—such as body mass index, weight loss, mobility, and self-perceived health—thus capturing systemic nutritional well-being beyond dietary intake alone [25]. Accordingly, the MNA serves as a plausible intermediary linking dietary patterns to cognitive outcomes, allowing examination of the pathway through generalized nutritional status.

#### 2.2.4. Covariates

In selecting covariates, this study was guided by the theoretical framework of SDoH [22]. A multi-level, systematic analytical approach was adopted to examine the structural and environmental underpinnings of individual health. The covariates encompass four key dimensions: sociodemographic and socioeconomic factors, health-related behaviors, health status and functional capacity, and subjective perceptions of the physical and social environment. This multidimensional approach was designed to rigorously control for potential confounding factors in the analysis of the association between hPDI and cognitive function, thereby allowing for a more precise estimation of its independent effect.

Sociodemographic and socioeconomic factors were assessed through the following measures: gender (male/female), age (in years, treated as a continuous variable), education level (grouped into three categories: no formal education, primary school, and junior high school or above), marital status (unmarried or married), monthly income (categorized into five bands: <¥1000, ¥1000–2000, ¥2001–5000, ¥5001–10,000, and >¥10,000), residential area (classified as city center, inner suburbs, or outer suburbs), and number of children (included as a continuous variable).

Health-related behaviors were captured through smoking status (classified as current smoker or non-smoker) and alcohol consumption (classified as current drinker or non-drinker). Physical activity was evaluated using the Physical Activity Scale for the Elderly (PASE), where a higher continuous score reflects a greater level of activity [26].

Health status and functional measures comprised three domains: chronic disease presence (yes/no), functional capacity, and depressive symptoms. Functional capacity was assessed using the Basic and Instrumental Activities of Daily Living scales (BADL and IADL) [27,28], where higher scores denote greater independence. Depressive symptoms were measured with Geriatric Depression Scale-15 (GDS-15) [29], analyzed as a continuous variable.

The subjective perceptions of the living environment were measured across three domains [30]—Housing, Transportation, and Community—using corresponding subscales adapted from the Age-friendly Community Evaluation Scale. Each item was rated on a 5-point Likert scale from 1 (“strongly disagree”) to 5 (“strongly agree”). All items were positively scored except for two housing-related statements—“I find housing-related expenses burdensome” and “My current home has fall risks and I wish to modify it”—which were reverse-coded (1 = 5, 2 = 4, 3 = 3, 4 = 2, 5 = 1). A total domain score was computed by summing the item scores within each domain, with higher scores indicating greater perceived satisfaction in that environmental dimension.

### 2.3. Statistical Analysis

First, descriptive analyses were conducted. Participant characteristics were summarized for the overall sample and further stratified by residential area. Categorical variables are presented as frequencies (percentages), and normally distributed continuous variables are expressed as mean ± standard deviations. Group differences across residential areas were examined using chi-square tests for categorical variables and one-way ANOVA for continuous variables.

Second, bivariate correlational analyses were performed to assess the bivariate relationships among all study variables. Pearson’s correlation coefficient was applied for continuous variables, while Spearman’s rank-order correlation was used for the ordinal CDR variable and other relevant ordinal covariates.

Third, hierarchical regression analyses were conducted to evaluate the independent association of the hPDI with each cognitive outcome. Hierarchical linear regression was used for continuous outcomes (MMSE, MoCA), and hierarchical ordinal logistic regression was applied for the ordinal outcome (CDR), respectively. The sequential inclusion of covariates in the hierarchical models was guided by the SDoH framework, reflecting a logical progression from distal to proximal influences on cognitive function. We first conducted null models (Model 1/7/13), then adjusted for sociodemographic factors (Model 2/8/14) as these represent fundamental personal and social characteristics. Socioeconomic status (income) was then added (Model 3/9/15) to account for resource-related determinants. Subsequently, health status indicators (chronic disease, functional capacity, depressive symptoms) were included (Model 4/10/16), as they directly relate to both dietary patterns and cognitive outcomes. Health behaviors (smoking, alcohol use, physical activity) were entered next (Model 5/11/17) given their potential to confound or mediate diet–cognition associations. Finally, perceived environmental factors were incorporated (Model 6/12/18) to capture contextual influences on lifestyle and access to healthy food. This stepwise approach allows examination of how the association between hPDI and cognitive outcomes is progressively shaped by different layers of potential confounding.

Fourth, to explore the potential interactive effects of the hPDI, gender, and residential area on cognitive function, hierarchical regression models incorporating interaction terms were applied. Separate analyses were conducted for each cognitive outcome: hierarchical linear regression for the continuous outcomes (MMSE, MoCA) and hierarchical ordinal logistic regression for the ordinal outcome (CDR). For each outcome, three progressively adjusted models were specified: the first model (Model A/D/G) included only the main effects of hPDI, gender, and residential area; the second model (Model B/E/H) added all two-way interaction terms (hPDI × Gender, hPDI × Area, Gender × Area); and the third model (Model C/F/K) further incorporated the three-way interaction term (hPDI × Gender × Area). All models were adjusted for the full set of covariates used in the primary analysis. The improvement in model fit attributable to the interaction terms was assessed: for linear models, the change in the coefficient of determination (ΔR^2^) was reported, and for ordinal logistic models, the change in Pseudo R^2^ was reported.

Fifth, a multi-step mediation analysis was performed to examine whether the association between the hPDI and cognitive function was statistically mediated by the MNA. First, the potential mediating role of MNA in the relationship between hPDI and each cognitive outcome was tested in the full sample, adjusting for all covariates. Subsequently, this mediation analysis was stratified by residential area (city center, inner suburbs, outer suburbs). Finally, to investigate potential heterogeneity further, the analysis was also stratified by both gender and residential area. Given the different measurement scales of the outcomes, the mediation models for the continuous MMSE and MoCA scores were conducted using ordinary least squares path analysis. In contrast, a path analysis framework employing a logistic link function—as recommended for ordinal outcomes—was applied for the CDR. In all mediation analyses, the total, direct, and indirect effects were estimated. The significance of the indirect (mediated) effect was evaluated using bias-corrected bootstrap confidence intervals derived from 5000 resamplings. It should be noted that this analysis is based on observational data and does not imply causal inference.

Finally, to address concerns about potential conceptual confounding arising from overlapping dietary content between the hPDI and the dietary intake components of the MNA, a sensitivity analysis was conducted. A modified MNA score (mMNA) was derived by excluding the two food and fluid intake items from the original MNA and summing the scores of the remaining items, thereby representing nutritional status independent of recent dietary intake. All multi-step mediation analyses described in the previous section were repeated using this mMNA as the mediator to evaluate the robustness of the observed mediation effects. All statistical analyses were conducted using Stata version 18.0 (StataCorp, College Station, TX, USA). Statistical significance was defined as a two-sided *p* value < 0.05.

Of note, this study includes both confirmatory and exploratory analyses. The hierarchical regressions examining the association between hPDI and cognitive outcomes, along with the full-sample mediation analysis through MNA, are confirmatory and test pre-specified hypotheses. In contrast, analyses involving interaction terms—both two-way and three-way—and mediation models stratified by area and gender are exploratory and aimed at generating new hypotheses. In statistical inference, we balanced the risks of Type I (false positive) and Type II (false negative) errors [31,32]. For confirmatory analyses, the a priori hypotheses clarify the inferential risks. For exploratory analyses—particularly underpowered tests of interactions and subgroup effects—overly conservative multiplicity corrections such as the Bonferroni method would unduly increase Type II error risk, potentially masking patterns of theoretical and public health relevance. Hence, uncorrected *p*-values are reported.

## 3. Results

### 3.1. Basic Characteristics

The baseline characteristics of the total sample (*N* = 2076), stratified by geographic area, are presented in Table 1. Significant disparities were observed among residents of the city center, inner suburbs, and outer suburbs. Participants residing in the city center generally exhibited more favorable sociodemographic profiles, including higher education attainment and income levels, compared to those in the outer suburbs, who presented the least favorable profiles. Health behaviors and status also differed significantly across areas, with the highest prevalence of chronic diseases observed in the city center, likely reflecting better detection due to superior healthcare access—a pattern consistent with urban health research [33]. The satisfaction of living environment was significantly lower in the outer suburbs and showed a progressive increase toward the city center. Notably, a clear geographic gradient was evident for key study variables: nutritional status (MNA), diet quality (hPDI), and all cognitive function scores (MMSE, MoCA, CDR) were most favorable in the city center and least favorable in the outer suburbs (all *p* < 0.05).

### 3.2. Results of Correlation Analysis

The bivariate correlations among the primary study variables are summarized in Table 2. A positive correlation was observed between the hPDI and the MNA score (r = 0.523, *p* < 0.001). Higher hPDI scores were significantly associated with better cognitive performance, evidenced by positive correlations with the MMSE (r = 0.380, *p* < 0.001) and MoCA (r = 0.314, *p* < 0.001) scores, and a negative correlation with the CDR (r = −0.318, *p* < 0.001). Similarly, better nutritional status (MNA) was positively correlated with both MMSE (r = 0.414, *p* < 0.001) and MoCA (r = 0.398, *p* < 0.001) scores, and negatively correlated with the CDR (r = −0.409, *p* < 0.001). As anticipated, the two global cognitive screening instruments, MMSE and MoCA, were strongly correlated with each other (r = 0.769, *p* < 0.001).

### 3.3. Results of Regression Analysis

The independent association between the hPDI and cognitive function, assessed through hierarchical regression analyses, is presented in Table 3.

A consistent and statistically significant association was found between the hPDI and all cognitive measures. In the unadjusted models, a higher hPDI score was significantly associated with better cognitive performance, reflected by higher scores on the MMSE (β = 0.160, *p* < 0.001) and MoCA (β = 0.136, *p* < 0.001), and a lower odds of a worse Clinical Dementia Rating (OR = 0.932, *p* < 0.001). These associations remained significant after sequential adjustment for a comprehensive set of potential confounders, including sociodemographic and socioeconomic factors, health status, health behaviors, and environmental factors.

While the strength of the associations attenuated with the inclusion of covariates—particularly following adjustment for health status (e.g., chronic disease, functional capacity, and depressive symptoms)—the hPDI remained a significant independent predictor of all cognitive outcomes in the fully adjusted models (MMSE: β = 0.083, *p* < 0.001; MoCA: β = 0.069, *p* < 0.001; CDR: OR = 0.944, *p* < 0.001). The variance explained by the linear regression models increased substantially with each added block of covariates. The final models explained 50.1% and 41.5% of the variance in MMSE and MoCA scores, respectively. Similarly, the model fit for the ordinal logistic regression of the CDR improved progressively with the sequential addition of covariates.

### 3.4. Results of Interaction Analysis

In the analysis of effect modification, this study examined whether gender and residential area moderate the association between hPDI and cognitive function. The significance of changes in the coefficient of determination (ΔR^2^) was used to evaluate the overall contribution of interaction terms. Hierarchical regression models were fitted with sequential inclusion of two-way (hPDI × gender, hPDI × area, gender × area) and three-way (hPDI × gender × area) interaction terms, adjusting for all covariates. The addition of the two-way interaction terms significantly improved model fit only for the MMSE (ΔR^2^ = 0.028, *p* < 0.001), but not for the MoCA or CDR. Furthermore, including the three-way interaction term further increased the explained variance for the MMSE (ΔR^2^ = 0.009, *p* < 0.001) and MoCA (ΔR^2^ = 0.003, *p* < 0.01), and led to a marginal yet significant improvement for the CDR model (ΔR^2^ = 0.001, *p* < 0.05). These results suggest that the relationship between hPDI and cognitive performance varies across subgroups defined by gender and residential area. Accordingly, the presentation in this section highlights ΔR^2^ as the primary indicator of interaction effects, while detailed estimates (β or OR) for individual interaction terms are provided in Table 4.

### 3.5. Results of Mediation Analysis

**Figure 1:**
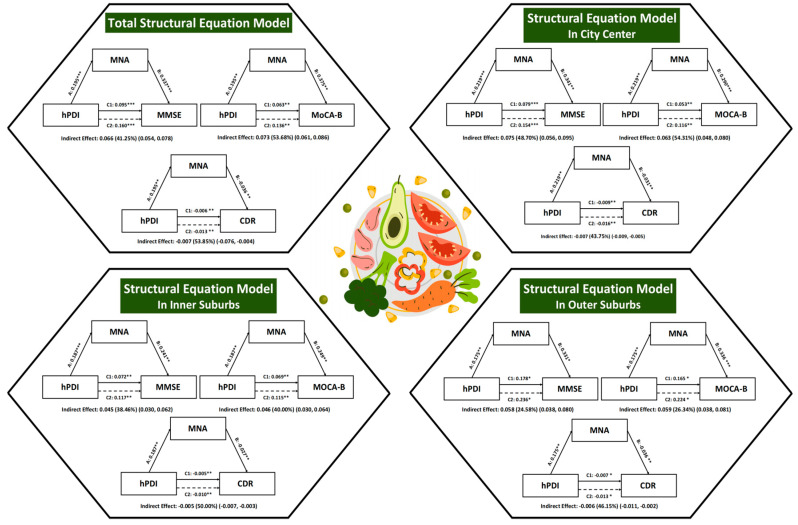
Mediation Pathways from the hPDI to Cognitive Function (MMSE, MoCA-B, CDR) Through the MNA Across Geographic Regions. Note. 1. All models controlled for age, education, marital status, number of children, income, chronic diseases, BADL, IADL, depression, smoking, drinking, PASE, and environmental factors. 2. BADL (Basic Activities of Daily Living); IADL (Instrumental Activities of Daily Living); PASE (Physical Activity Scale for the Elderly); MNA (Mini Nutritional Assessment); hPDI (Healthful Plant-based Diet Index); MMSE (Mini-Mental State Examination); MoCA-B (Montreal Cognitive Assessment-Basic); CDR (Clinical Dementia Rating). ***: *p* < 0.001; **: *p* < 0.01; *: *p* < 0.05.

A multi-stage mediation analysis was conducted to assess whether nutritional status (MNA) mediated the association between the hPDI and cognitive function. The analysis was performed first in the overall sample and subsequently stratified by geographic area (see Figure 1, clear version provided in Supplementary Material File) and further by both area and gender (Table 5).

In the total sample, MNA significantly mediated the relationship between hPDI and all cognitive outcomes. The indirect effects were statistically significant, accounting for 41.25%, 53.68%, and 53.85% of the total effect of hPDI on MMSE, MoCA-B, and CDR, respectively.

Notably, the strength and significance of these mediation pathways showed considerable heterogeneity across geographic areas and by gender. When stratified jointly by area and gender, distinct patterns emerged. The mediating effect of MNA was generally strongest and most consistent among participants in the city center, for both males and females. For example, in the city center, the proportion mediated for the MoCA-B was 68.54% in males and 47.73% in females.

Mediation patterns varied in other subgroups. In the inner suburbs, the total effect of hPDI on cognitive function was non-significant among males across MMSE, MoCA-B, and CDR. In the outer suburbs, significant associations were primarily observed in females, for whom the total, direct, and indirect effects on MMSE, MoCA-B, and CDR were all significant; MNA mediated 20.54%, 37.62%, and 23.46% of these effects, respectively. By contrast, most associations were non-significant among males in the outer suburbs.

In summary, nutritional status is a significant mediator of the association between hPDI and cognitive performance. However, the completeness and strength of this potential mediation pathway are not uniform and are influenced by a complex interplay between geographic area and gender, with the most robust and consistent effects observed in the city center and among females in the suburban areas.

### 3.6. Results of Sensitive Analysis

To address potential conceptual overlap between the hPDI and the dietary components of the MNA, a sensitivity analysis was performed using a mMNA score from which all diet-related items were excluded. The results substantiated the robustness of our primary findings. In the total sample, the indirect associations of hPDI with all cognitive measures (MMSE, MoCA-B, CDR) through mMNA remained statistically significant (*p* < 0.01). The geographic patterns of mediation were largely preserved, with mMNA explaining a substantial proportion (e.g., >60% for MMSE and MoCA-B in the city center and inner suburbs) of the total association. Furthermore, the key subgroup pattern identified in the primary analysis—namely, that the association in the outer suburbs was primarily evident among females—was successfully replicated using the mMNA. Detailed results are provided in the Supplementary Materials.

## 4. Discussion

Through a multi-stage stratified sampling survey involving 2079 older adults in Shanghai, this study systematically examined the relationship between the hPDI and multidimensional cognitive function, elucidated the potential mediating role of the MNA, and further investigated the heterogeneity of this relationship across urban–suburban regions and gender groups. The findings contribute to and extend the existing body of evidence at multiple levels, both within China and outside China.

### 4.1. The hPDI–Cognition Association: Consistent Evidence and Validation in China

This study, conducted among older adults in Shanghai—a representative megacity in China—confirms a significant protective association between higher hPDI scores and cognitive function, consistent with recent evidence. For instance, analyses from the Rotterdam Study cohort indicated that a higher hPDI score was independently associated with a reduced risk of dementia in men (adjusted HR = 0.86, 14% risk reduction) and among APOE ε4 carriers (adjusted HR = 0.83, 17% risk reduction) after multivariable adjustment, collectively underscoring the central role of plant-based food quality in neuroprotection [34]. Furthermore, by utilizing a multidimensional cognitive assessment system—including the MMSE (global cognition), MoCA-B (executive function), and CDR (clinical staging)—this research not only validates the robustness of the hPDI–cognition link within the Chinese context but also supports the generalizability of diet–cognitive associations across distinct cognitive domains and clinical stages.

### 4.2. The Potential Mediating Role of Nutritional Status: Linking Dietary Patterns to Cognitive Function

In this cross-sectional analysis, MNA was identified as a significant mediator in the relationship between hPDI and cognitive function, pointing to a potential “diet-nutrition-cognition” pathway that merits longitudinal investigation (it should be noted that, due to the limitations of the cross-sectional design, this study cannot infer the directionality of this mediation effect). This observation aligns with micro-level mechanisms reported in Western studies—such as neuroinflammation inhibition, oxidative stress mitigation, and gut–brain axis modulation [35,36]. Adequate overall nutritional status is recognized as a systemic basis for maintaining inflammatory balance and antioxidant capacity. Thus, the mediating role of MNA may offer a theoretical link between measurable nutritional indicators and underlying probable biological mechanisms [37].

Within the China’s research context, where emphasis has often been placed on single-nutrient associations with cognition (e.g., folate, vitamin B12) [38], this study extends the perspective toward overall dietary patterns and integrated nutritional status. It suggests that enhancing overall nutritional quality-rather than focusing solely on isolated nutrients-could provide broader public health benefits in slowing cognitive decline, reflecting a shift toward combining holistic and precision nutrition approaches.

Notwithstanding these consistent findings, the cross-sectional design necessitates caution regarding causal inference. Reverse causality cannot be excluded: poorer cognitive function may itself lead to dietary alterations—such as reduced intake of healthful plant-based foods—rather than the converse. Individuals with cognitive impairment or early dementia may experience alterations in appetite, food preference, meal-preparation capacity, or social eating patterns, which could result in lower hPDI scores and compromised nutritional status (MNA). Although we adjusted for a comprehensive set of covariates—such as functional capacity (BADL/IADL) and depressive symptoms—residual confounding from unmeasured or imprecisely measured factors related to cognitive decline and dietary behavior remains plausible. Thus, our findings should be interpreted as statistical associations that underscore a link between diet, nutrition, and cognitive outcomes, not as evidence of causal direction. Longitudinal or interventional studies are warranted to determine whether enhancing plant-based diet quality can indeed attenuate cognitive decline in older adults.

### 4.3. Heterogeneity Across Cognitive Measures: Context-Dependent Interactions Between Diet, Area, and Gender

Our analysis of interaction terms revealed notable heterogeneity across cognitive assessment tools. The MMSE, as a macro-level screening instrument, consistently captured both the direct association of hPDI with cognitive function and the full statistical significance of all two-way and three-way interactions involving hPDI, geographic area, and gender. In contrast, more precise, domain-specific scales-namely the MoCA-B (assessing higher-order cognition) and the CDR (evaluating clinical staging)—showed a different pattern: while the three-way interaction (hPDI × area × gender) remained significant, the specific two-way interactions (“hPDI × gender” and “hPDI × area”) were not statistically prominent.

These findings demonstrated that the properties of the cognitive assessment tool critically shaped the observed relationship between hPDI and cognitive function. The interacting role of gender, in particular, appears to depend on the specific cognitive domain or stage being measured. This underscores the unique value of precise scales: at the level of higher-order cognition and clinical staging, geographic and gender factors may operate as an inseparable contextual system [39]. Within such a system, the association of an hPDI with cognitive outcomes is best understood through this integrated three-way lens, rather than through isolated binary interactions. Informed by this insight, we conducted subsequent stratified analyses by area and gender, which yielded more targeted findings.

### 4.4. Contextualizing Diet–Cognition Association: The Interplay of Geography, Gender, and Socio-Behavioral Factors

This study observed no significant regional heterogeneity in the association between the hPDI and cognitive function across central urban, inner suburban, and outer suburban areas of Shanghai. This finding contrasts with common urban–rural health disparities documented in Europe and the United States, which are often correlated with structural inequities such as “food deserts” [40]. The consistent association observed across all regions may be linked to Shanghai’s integrated urban governance, which includes a comprehensive logistics network, continuous improvements to the “Vegetable Basket Project” [41], and advancing urban–rural integration—factors that collectively promote equitable access to healthful plant-based foods. A key empirical observation was the significant three-way interaction (hPDI × Gender × Area), reflected in a non-significant association specifically among male residents in suburban areas. This pattern highlights the contextual nature of diet–cognition links and aligns partially with Mediterranean population studies, where the health associations of dietary patterns such as the Mediterranean diet have often been more evident among women [42,43]. Together, these results provide a relevant case for considering region-wide dietary strategies in other global megacities with comparable infrastructure.

To explain the lack of association between an hPDI and cognitive benefit among suburban males, we propose the speculative “triple disadvantage” hypothesis. This framework posits that the null association may stem from interconnected disadvantages at dietary, behavioral, and sociocultural levels, extending beyond diet alone.

Gender disparities in dietary patterns were evident. Older women tended to consume lighter, more varied diets focused on natural flavors and nutritional balance, often related to self-care [44]. In contrast, men were more likely to follow traditional local diets high in salt and fat and low in fresh vegetables [45]. Even when plant foods were eaten, preparation methods such as frying, salting, or pickling—common in regional cuisine—may reduce nutrient bioavailability and align with an unhealthful plant-based pattern.

Marked gender differences emerged in health behaviors. Older suburban men reported higher rates of smoking and alcohol use, along with lower participation in structured health management and community activities [46]. These concurrent risk factors may offset potential cognitive benefits from diet. Conversely, women showed greater engagement in social and physical activities that reinforce healthier lifestyles [47].

Food choices are embedded in gendered identities. Diets richer in animal fats, salt, and meat are often viewed as markers of masculinity, shaped by social expectations [48]. Conversely, “lighter” or plant-predominant diets may be perceived as less compatible with traditional masculine roles, potentially reducing adherence and psychological receptivity, thereby weakening the diet–cognition link.

## 5. Implications and Generalizability

Conducted within the specific context of Shanghai—a city with a highly integrated food supply and governance system—this study found no geographic gradient in the association between the hPDI and cognitive function. This pattern may be related to the equitable food environment shaped by policies such as the “Vegetable Basket Project.” Therefore, the result is highly context-dependent, and its direct generalizability to other regions lacking comparable comprehensive food-policy systems may be limited.

On the other hand, insights derived from mechanistic and subgroup analyses hold broader relevance. First, the mediating role of overall nutritional status in the diet–cognition relationship offers a transferable framework that can be examined across diverse settings. Second, the observed interaction between gender and residential area—for instance, the absence of a significant association among suburban males—generally suggests that diet-health relationships are likely moderated by sociocultural and behavioral contexts, rather than reflecting a uniform or deterministic effect.

In summary, although the observed association between diet and cognition was modest at the individual level (β ≈ 0.07–0.08), diet remains a modifiable lifestyle factor with potential cumulative protective effects that are of public health importance. To advance cognitive health equitably, future strategies should extend beyond promoting dietary quality alone and instead adopt integrated, contextually designed interventions that incorporate local food practices, related health behaviors, and gender norms—particularly for subgroups that may show limited response to dietary changes alone.

## 6. Conclusions

This cross-sectional study documents a systematic association between higher hPDI scores and better cognitive function, mediated in part by improved nutritional status (MNA). Using Shanghai as a case study, it revealed—for the first time in a non-Western megacity—that this protective association remains geographically homogeneous, a pattern attributable to integrated food supply systems and coordinated urban–rural governance that help mitigate structural inequalities in food access.

However, a significant gender-by-area interaction indicates that older men in suburban areas derived no cognitive benefit from higher hPDI. This pattern may reflect a plausible constellation of factors: their dietary patterns appear more anchored in traditional local diets, which tend to be higher in salt and fat and prepared using less healthy methods; they also exhibit higher rates of behavioral risks such as smoking and alcohol consumption, alongside lower engagement in preventive health management. Furthermore, socio-cultural perceptions in these contexts may view healthy eating as less compatible with local masculine identity, potentially limiting its adoption and sustainability. Thus, public health interventions should move beyond promoting healthy diets alone and adopt socio-culturally embedded strategies. While maintaining equitable food access across regions, tailored approaches—particularly in suburban settings—could include developing nutritionally improved recipes that preserve local taste preferences and align with masculine identity, integrated with smoking cessation, alcohol reduction, and community health initiatives designed to engage men. Such integrated efforts may help disrupt the reinforcing cycle linking diet, behavior, and gender norms, enabling dietary improvements to benefit all subgroups equitably.

## 7. Strengths and Limitations

This study provides novel evidence by documenting both the equitable geographic distribution of the diet–cognition association within a uniquely governed non-Western megacity and its significant sociocultural exception among suburban males, thereby advancing a dual-perspective framework for precision public health that integrates systemic food policy with context-sensitive behavioral intervention.

This study has several limitations that warrant careful consideration. First, its cross-sectional design precludes causal inference between dietary patterns, nutritional status, and cognitive outcomes; reverse causation and residual confounding remain plausible. Second, key explanatory mechanisms—such as gender-specific cooking practices and sociocultural norms—were not directly measured, rendering our interpretations speculative. Simultaneously, the reliance on self-reported data (e.g., FFQ) is also susceptible to measurement error and recall bias. Future studies should employ mixed-methods approaches, integrating enhanced quantitative tools and qualitative interviews with objective measures like dietary photography and nutritional biomarkers (e.g., blood carotenoids) to validate speculative mechanisms and self-reported dietary data. Third, the findings are derived from Shanghai, a uniquely developed megacity with a robust food supply system, which limits generalizability to rural or structurally disadvantaged settings. Fourth, despite hypothesis-driven analyses using robust metrics (e.g., ΔR^2^, bootstrap CIs), the multiple statistical tests conducted across outcomes, models, and strata may increase the risk of Type I error. Therefore, the interaction and subgroup findings—especially those related to suburban males—should be interpreted as preliminary and considered hypothesis-generating. Further validation through longitudinal or intervention-based studies in independent samples is warranted.

## Figures and Tables

**Table 1:** Comparison of demographic characteristics across three residential tiers (city center, inner suburban, and outer suburban).

Variable	Total	City Center	Inner Suburbs	Outer Suburbs	F/χ^2^
Gender, *n* (%)					7.829 *
male	820 (39.44)	295 (40.75)	228 (35.08)	297 (42.13)	
female	1259 (60.56)	429 (59.25)	422 (64.92)	408 (57.87)	
Age, Mean ± SD	72.61 ± 6.52	72.28 ± 6.21	72.08 ± 6.84	73.43 ± 6.46	20.160 ***
Education, *n* (%)					281.142 ***
No formal education	210 (10.10)	35 (4.84)	57 (8.77)	118 (16.74)	
primary school	580 (27.90)	83 (11.46)	199 (30.62)	298 (42.47)	
Junior high school and above	1289 (62.00)	606 (83.70)	394 (60.62)	289 (40.99)	
Marital status, *n* (%)					3.938
Unmarried	438 (21.07)	145 (20.03)	154 (23.69)	139 (19.72)	
Married	1641 (78.93)	579 (79.97)	496 (76.31)	566 (80.28)	
Monthly income, *n* (%)					920.384 ***
<1000	35 (1.68)	8 (1.10)	9 (1.38)	18 (2.56)	
1000–2000	273 (13.13)	6 (0.83)	18 (2.77)	249 (35.32)	
2001–5000	1235 (59.40)	303 (41.85)	539 (82.92)	393 (55.74)	
5001–10,000	492 (23.67)	380 (52.49)	79 (12.15)	33 (4.68)	
>10,000	44 (2.12)	27 (3.73)	5 (0.77)	12 (1.70)	
Number of children, *n* (%)					209.986 ***
0	33 (1.59)	23 (3.18)	4 (0.62)	6 (0.85)	
1	1259 (60.56)	558 (77.07)	346 (53.23)	355 (50.35)	
2	653 (31.41)	116 (16.02)	255 (39.23)	282 (40.00)	
3	112 (5.39)	19 (2.62)	40 (6.15)	53 (7.52)	
≥4	22 (1.06)	8 (1.11)	5 (0.77)	9 (1.27)	
Chronic disease, *n* (%)					435.199 ***
Yes	960 (46.18)	614 (84.81)	385 (59.23)	465 (65.96)	
No	1119 (53.82)	110 (15.19)	265 (40.77)	240 (34.04)	
BADL, Mean ± SD	98.80 ± 4.8 5	98.14 ± 5.88	98.71 ± 4.39	99.57 ± 3.89	16.672 ***
IADL, Mean ± SD	8.28 ± 1.43	8.39 ± 1.27	8.29 ± 1.56	8.16 ± 1.45	17.022 ***
Depression, Mean ± SD	3.95 ± 3.22	3.05 ± 3.19	4.06 ± 3.15	4.77 ± 3.09	129.650 ***
MNA, Mean ± SD	25.16 ± 3.59	25.64 ± 3.58	25.52 ± 3.62	24.33 ± 3.41	93.172 ***
MMSE, Mean ± SD	24.77 ± 4.07	25.93 ± 3.07	24.41 ± 3.79	23.92 ± 4.87	82.252 ***
MoCA-B, Mean ± SD	23.55 ± 4.18	25.30 ± 2.63	23.57 ± 3.58	21.74 ± 5.11	286.182 ***
CDR, Mean ± SD	0.24 ± 0.39	0.12 ± 0.25	0.26 ± 0.39	0.34 ± 0.46	88.627 ***
Smoke, *n* (%)					35.325 ***
Yes	347 (16.69)	73 (10.08)	136 (20.92)	138 (19.57)	
No	1732 (83.31)	651 (89.92)	514 (79.08)	567 (80.43)	
Alcohol, *n* (%)					435.199 ***
Yes	230 (11.06)	114 (15.75)	53 (8.15)	63 (8.94)	
No	1849 (88.94)	610 (84.25)	597 (91.85)	642 (91.06)	
PASE, Mean ± SD	136.89 ± 51.58	139.40 ± 55.95	133.47 ± 49.31	137.53 ± 49.00	10.301 **
hPDI, Mean ± SD	56.23 ± 9.62	56.91 ± 8.46	56.04 ± 9.03	55.75 ± 11.07	6.013 *
Environmental satisfaction, Mean ± SD	58.87 ± 11.35	56.18 ± 11.14	58.96 ± 10.88	61.56 ± 11.36	86.710 ***

Note: BADL (Basic Activities of Daily Living); IADL (Instrumental Activities of Daily Living); PASE (Physical Activity Scale for the Elderly); MNA (Mini Nutritional Assessment); hPDI (Healthful Plant-based Diet Index); MMSE (Mini-Mental State Examination); MoCA-B (Montreal Cognitive Assessment-Basic); CDR (Clinical Dementia Rating). ***: *p* < 0.001; **: *p* < 0.01; *: *p* < 0.05.

**Table 2:** Correlations between hPDI, MNA, and Cognitive Function (MMSE, MoCA-B, CDR).

Variable	1	2	3	4	5
1 hPDI	1				
2 MNA	0.523 ***	1			
3 CDR	−0.318 ***	−0.409 ***	1		
4 MoCA-B	0.314 ***	0.398 ***	−0.679 ***	1	
5 MMSE	0.380 ***	0.414 ***	−0.592 ***	0.769 ***	1

Note: ***: *p* < 0.001. MNA (Mini Nutritional Assessment); hPDI (Healthy Plant-based Diet Index); MMSE (Mini-Mental State Examination); MoCA-B (Montreal Cognitive Assessment-Basic); CDR (Clinical Dementia Rating).

**Table 3:** Hierarchical Regression Analysis of the Association Between the hPDI and Cognitive Function, Assessed Across MMSE, MoCA-B, and CDR in Sequentially Adjusted Models.

MMSE			MoCA-B			CDR		
Model	β (95% CI)	R^2^	Model	β (95% CI)	R^2^	Model	OR (95% CI)	R^2^
Model 1	0.160 *** (0.144, 0.177)	0.144	Model 7	0.136 *** (0.118, 0.154)	0.098	Model 13	0.932 *** (0.923, 0.940)	0.065
Model 2	0.123 *** (0.109, 0.138)	0.424	Model 8	0.104 *** (0.088, 0.119)	0.310	Model 14	0.938 *** (0.929 0.947)	0.090
Model 3	0.123 *** (0.109, 0.137)	0.435	Model 9	0.103 *** (0.087, 0.118)	0.345	Model 15	0.933 *** (0.925, 0.943)	0.118
Model 4	0.092 *** (0.077, 0.106)	0.484	Model 10	0.077 *** (0.062, 0.093)	0.393	Model 16	0.948 *** (0.938, 0.958)	0.176
Model 5	0.083 *** (0.069, 0.097)	0.501	Model 11	0.069 *** (0.053, 0.084)	0.414	Model 17	0.952 *** (0.942, 0.962)	0.191
Model 6	0.083 *** (0.069, 0.097)	0.501	Model 12	0.069 *** (0.053, 0.085)	0.415	Model 18	0.944 *** (0.934, 0.955)	0.205

Note: ***: *p* < 0.001. Models 1, 7, 13 (MMSE, MoCA-B, CDR, respectively): Crude model; Models 2, 8, 14 (MMSE, MoCA-B, CDR, respectively): +(Age, Gender, Education, Marital status, Number of children); Models 3, 9, 15 (MMSE, MoCA-B, CDR, respectively): +(Income); Models 4, 10, 16 (MMSE, MoCA-B, CDR, respectively): +(Chronic disease, BADL, IADL, Depression); Models 5, 11, 17 (MMSE, MoCA-B, CDR, respectively): +(Smoke, Alcohol, PASE); Models 6, 12, 18 (MMSE, MoCA-B, CDR, respectively): +(Environmental factors).

**Table 4:** Hierarchical Regression Analysis of hPDI and Cognitive Function Across Three Measures: Moderating Roles of Gender and Residential Area.

Variable	MMSE (β)			MoCA-B (β)			CDR (OR)		
	Model A	Model B	Model C	Model D	Model E	Model F	Model G	Model I	Model H
hPDI	0.082 ***	−0.020	0.036 *	0.074 ***	−0.030 *	−0.008	0.944 ***	0.939 ***	0.926 ***
Gender Ref: Female	−0.181	−4.861 ***	−0.004	−0.068	−3.886 ***	−0.191	1.119	1.353	1.059
Area (Inner Suburbs) Ref: City Center	−0.105	−1.344	3.577 *	−0.425 *	−3.268 **	−2.751	2.079 ***	0.038 ***	0.022 ***
Area (Outer Suburbs) Ref: City Center	0.223	−7.85 ***	−0.718	−1.526 ***	0.160 ***	−2.39 ***	3.539 ***	0.221	0.033 **
hPDI × Gender Ref: Female		0.084 ***	−0.002 *		0.067	0.032		0.943	0.966
hPDI × Area (Inner Suburbs) Ref: City Center		0.027 *	−0.061 *		0.053 *	0.044 *		1.075	1.088
hPDI × Area (Outer Suburbs) Ref: City Center		0.14 ***	0.015 *		0.160	0.084		1.052	1.090
Gender × Area (Inner Suburbs) Ref: Female, City Center		−0.442 *	−8.086 ***		−0.191	−1.107		1.696	4.462
Gender × Area (Outer Suburbs) Ref: Female, City Center		0.216 *	−10.815 **		0.342	0.084		1.462	2.720
hPDI × Gender × Area (Inner Suburbs) Ref: Female, City Center			0.137 ***			0.016 **			1.979 **
hPDI × Gender × Area (Outer Suburbs) Ref: Female, City Center			0.194 ***			0.119 **			1.946 **
R^2^/Pseudo R^2^	0.508	0.536	0.545	0.440	0.467	0.470	0.205	0.221	0.222
Adjusted R^2^	0.503	0.530	0.538	0.434	0.460	0.462	-	-	-
∆R^2^	-	0.028 ***	0.009 ***	-	0.027	0.003 **	-	0.016	0.001 **

Note: 1. All models controlled for age, education, marital status, number of children, income, chronic diseases, BADL, IADL, depression, smoking, drinking, PASE, and environmental factors. 2. For each cognitive measure, Model A/D/G (MMSE, MoCA-B, CDR, respectively) included main effects; Model B/E/I (MMSE, MoCA-B, CDR, respectively) added two-way interactions; Model C/F/H (MMSE, MoCA-B, CDR, respectively) added the three-way interaction. Area (city center, inner suburb, outer suburb) and gender (male, female) were categorical variables. 3. BADL (Basic Activities of Daily Living); IADL (Instrumental Activities of Daily Living); PASE (Physical Activity Scale for the Elderly); MNA (Mini Nutritional Assessment); hPDI (Healthful Plant-based Diet Index); MMSE (Mini-Mental State Examination); MoCA-B (Montreal Cognitive Assessment-Basic); CDR (Clinical Dementia Rating). 4. ***: *p* < 0.001; **: *p* < 0.01; *: *p* < 0.05.

**Table 5:** The Mediating Role of the MNA in the Association Between hPDI and Cognitive Function (MMSE, MoCA-B, CDR) Across Geographic Area and Gender Strata.

Impact Pathway	Area	Gender	Total Effect	Direct Effect	Indirect Effect	Proportion	95% CI
hPDI→MMSE	City Center	Male	0.141 **	0.087 **	0.054 **	38.30%	0.032, 0.080
		Female	0.160 **	0.073 **	0.088 **	55.00%	0.061, 0.116
	inner suburbs	Male	0.145	0.091	0.054	37.24%	−0.020, 0.092
		Female	0.196 **	0.131 **	0.065 **	33.16%	0.045, 0.086
	outer suburbs	Male	0.060	0.033	0.027	45.00%	−0.008, 0.062
		Female	0.331 **	0.264 **	0.068 **	20.54%	0.045, 0.093
hPDI→MoCA-B	City Center	Male	0.089 **	0.028 *	0.061 **	68.54%	0.039, 0.086
		Female	0.132 **	0.068 **	0.063 **	47.73%	0.043, 0.086
	inner suburbs	Male	−0.139	−0.055	−0.084	60.43%	0.048, 0.124
		Female	0.160 **	0.113 **	0.047 **	29.38%	0.030, 0.065
	outer suburbs	Male	0.081	0.048	0.033	40.74%	−0.004, 0.119
		Female	0.303 **	0.235 **	0.114 **	37.62%	0.079, 0.155
hPDI→CDR	City Center	Male	−0.079 **	−0.022 **	−0.057 **	72.15%	−0.087, −0.056
		Female	−0.265 **	−0.191 **	−0.074 **	27.92%	−0.095, −0.053
	inner suburbs	Male	−0.065	−0.037	−0.028	43.08%	−0.053, −0.004
		Female	−0.051 **	−0.039 **	−0.012 **	23.53%	−0.017, −0.008
	outer suburbs	Male	−0.022	−0.018	−0.004	18.18%	−0.021, 0.013
		Female	−0.081 **	−0.062 **	−0.019 **	23.46%	−0.026, −0.012

Note. 1. All models controlled for age, education, marital status, number of children, income, chronic diseases, BADL, IADL, depression, smoking, drinking, PASE, and environmental factors. 2. BADL (Basic Activities of Daily Living); IADL (Instrumental Activities of Daily Living); PASE (Physical Activity Scale for the Elderly); MNA (Mini Nutritional Assessment); hPDI (Healthful Plant-based Diet Index); MMSE (Mini-Mental State Examination); MoCA-B (Montreal Cognitive Assessment-Basic); CDR (Clinical Dementia Rating). 3. **: *p* < 0.01; *: *p* < 0.05.

## Data Availability

Dataset available on request from the authors.

## Supplementary Materials

The following supporting information can be downloaded at: https://www.mdpi.com/article/10.3390/nu18020316/s1, Supplementary Table S1. Sampling locations by geographic stratum; Supplementary Table S2. Mediation Analysis of hPDI on Cognitive Function through the Modified MNA (mMNA), Stratified by Geographic Area; Supplementary Table S3. Mediation Analysis of hPDI on Cognitive Function through the Modified MNA (mMNA), Stratified by Geographic Area and Gender; Supplementary questionnaires.

## Abbreviations

The following abbreviations are used in this manuscript:

AD	Alzheimer’s disease
hPDI	healthful plant-based diet index
SDoH	social determinants of health
MNA	Mini Nutritional Assessment
MMSE	Mini-Mental State Examination
MoCA-B	Montreal Cognitive Assessment-Basic
CDR	Clinical Dementia Rating
FFQ	food frequency questionnaire
PASE	Physical Activity Scale for the Elderly
BADL	Basic Activities of Daily Living
IADL	Instrumental Activities of Daily Living
GDS-15	Geriatric Depression Scale-15
