# Supplementary material for: Healthful Plant-Based Diets and Cognitive Function in Older Adults: Mediation by Nutritional Status and Modification by Urban–Suburban Location and Gender in a Shanghai Community-Based Study
Source: Nutrients. 2026 Jan 19;18(2):316. doi: 10.3390/nu18020316 (PMC12845157; doi:10.3390/nu18020316)
Supplement: Supplementary file 1 [file nutrients-18-00316-s001.zip › nutrients-4069251-supplementary.pdf]

Supplementary Table S1. Sampling locations by geographic stratum。

Geographic Stratum	District	Subdistrict
**City Center**	Changning District	Beixinjing
	Xuhui District	Changqiao
**Inner Suburbs**	Jiading District	Juyuan
	Pudong New Area	Tangzhen
**Outer Suburbs**	Fengxian District	Zhuanghang
	Pudong New Area	Chuansha

To address the potential concern of conceptual overlap between the hPDI and the dietary components of the MNA, we conducted a comprehensive sensitivity analysis using a modified MNA score (mMNA) that excluded all diet-related items. The results of this analysis were substantively consistent with our primary findings, confirming the robustness of the mediating role of nutritional status.

As detailed in Supplementary Table S1, when stratifying by geographic area only, the indirect effects of hPDI on all three cognitive measures through mMNA remained statistically significant ($p < 0.01$) in the total sample and across all areas. The proportion of the total effect mediated by mMNA showed patterns highly similar to those observed with the full MNA, with notably strong mediation in the city centre and inner suburbs for MMSE and MoCA.

Furthermore, the more granular analysis stratified by both gender and geographic area (Supplementary Table S2) reinforced the key subgroup patterns identified in the primary analysis. The mediating effect of mMNA continued to be most pronounced and consistent in the city centre for both genders. Crucially, the finding that the association in the outer suburbs is primarily driven by females was replicated, with significant indirect effects through mMNA observed for females but not for males across all cognitive outcomes.

In summary, the persistence of significant mediation effects through the diet-independent mMNA score strongly indicates that the pathway from a healthful plant-based diet to better cognitive function via improved nutritional status is not an artifact of measurement overlap. Instead, it represents a robust relationship that encompasses broader, systemic aspects of

nutritional well-being beyond mere dietary intake frequency.

Supplementary Table S2. Mediation Analysis of hPDI on Cognitive Function through the Modified MNA (mMNA), Stratified by Geographic Area.

Impact Pathway	Area	Total effect	Direct effect	Indirect effect	Proportion	95% CI
**hPDI→MMSE**	Total	0.083**	0.041**	0.042**	50.60%	0.031, 0.052
	City Center	0.044**	0.019**	0.023**	69.35%	0.014, 0.036
	Inner suburbs	0.068**	0.025**	0.043**	63.24%	0.025, 0.061
	Outer suburbs	0.164**	0.115**	0.049**	29.88%	0.027, 0.072
**hPDI→MOCA**	Total	0.074**	0.034**	0.040**	54.05%	0.029, 0.052
	City Center	0.035**	0.007**	0.028**	80.00%	0.017, 0.039
	Inner suburbs	0.072**	0.029**	0.043**	60.72%	0.024, 0.062
	Outer suburbs	0.156**	0.108**	0.047**	30.32%	0.022, 0.073
**hPDI→CDR**	Total	-0.058**	-0.026**	-0.032**	55.17%	-0.040, -0.025
	City Center	-0.183**	-0.096**	-0.087**	47.54%	-0.110, -0.066
	Inner suburbs	-0.029**	-0.006**	-0.023**	79.31%	-0.036, -0.011
	Outer suburbs	-0.045**	-0.018**	-0.027**	60.00%	-0.040, -0.014

Note.

1. All models controlled for age, education, marital status, number of children, income, chronic diseases, BADL, IADL, depression, smoking, drinking, PASE, and environmental factors.
2. BADL (Basic Activities of Daily Living); IADL (Instrumental Activities of Daily Living); PASE (Physical Activity Scale for the Elderly); mMNA (modified Mini Nutritional Assessment); hPDI (Healthful Plant-based Diet Index); MMSE (Mini-Mental State Examination); MoCA-B (Montreal Cognitive Assessment-Basic); CDR (Clinical Dementia Rating).
3. ***: $p < 0.001$; **: $p < 0.01$; *: $p < 0.05$.

Supplementary Table S3. Mediation Analysis of hPDI on Cognitive Function through the Modified MNA (mMNA), Stratified by Geographic Area and Gender.

Impact Pathway	Area	Gender	Total effect	Direct effect	Indirect effect	Proportion	95% CI
**hPDI→MMSE**	City Centre	Male	0.051**	0.029**	0.022**	43.14%	0.008, 0.036
		Female	0.036**	0.014**	0.022**	61.11%	0.008, 0.037
	inner	Male	0.084	0.052	0.032	38.10%	-0.010, 0.073

	suburbs	Female	0.109**	0.058**	0.052**	47.27%	0.033, 0.070
	outer	Male	0.027	0.007	0.020	74.07%	-0.024, 0.064
	suburbs	Female	0.229**	0.176**	0.053**	23.14%	0.028, 0.078
**hPDI→MOCA**	City	Male	0.058**	0.030**	0.028**	48.28%	0.013, 0.043
	Centre	Female	0.045**	0.026*	0.019*	42.22%	0.003, 0.035
	inner	Male	0.128	0.052	0.076**	59.38%	0.029, 0.124
	suburbs	Female	0.100**	0.065**	0.034**	34.34%	0.016, 0.053
	outer	Male	0.054	0.026	0.027	50.94%	-0.028, 0.083
	suburbs	Female	0.200**	0.152**	0.048**	24.00%	0.021, 0.075
**hPDI→CDR**	City	Male	-0.100**	-0.017**	-0.083**	83.00%	-0.124, -0.042
	Centre	Female	-0.229**	-0.127**	-0.102**	44.54%	-0.135, -0.069
	inner	Male	-0.076	-0.043	-0.033*	43.42%	-0.067, -0.001
	suburbs	Female	-0.050**	-0.027**	-0.023**	46.00%	-0.035, -0.011
	outer	Male	-0.014	-0.019	-0.005	-35.71%	-0.033, 0.023
	suburbs	Female	-0.081**	-0.046**	-0.035**	43.21%	-0.049, -0.021

Note.

1. All models controlled for age, education, marital status, number of children, income, chronic diseases, BADL, IADL, depression, smoking, drinking, PASE, and environmental factors.

2. BADL (Basic Activities of Daily Living); IADL (Instrumental Activities of Daily Living); PASE (Physical Activity Scale for the Elderly); mMNA (modified Mini Nutritional Assessment); hPDI (Healthful Plant-based Diet Index); MMSE (Mini-Mental State Examination); MoCA-B (Montreal Cognitive Assessment-Basic); CDR (Clinical Dementia Rating).

3. ***: $p < 0.001$; **: $p < 0.01$; *: $p < 0.05$.

Dietary Information - Food frequency questionnaire					
How often do you consume the following foods?	Almost every day	Weekly≥1times	Monthly≥1times	Occasionally	Rarely or never
1.Whole grains					
2.Fruits					
3.Vegetables or vegetable juice					
4. Nuts					
5. Legumes and soy products					
6. Vegetable oil					
7. Tea					
8. Coffee					
9. Pure squeezed fruit juice					
10. Sugary drinks					
11. Refined grains					
12. Potato products (such as fries, mashed potatoes, potato chips)					
13. Candy or desserts					
14. Animal fats (such as butter, lard)					
15. Milk or dairy products (cream, ice cream, etc.)					
16. Eggs					
17. Fish or seafood					
18. Meat					
18-1 Red meat (such as beef)					
18-2 White meat (such as					
19-ther animal-based foods like pizza					

DeepL

Subscribe to DeepL Pro to translate larger documents.
Visit www.DeepL.com/pro for more information.

Long MNA®
Mini Nutritional Assessment

Nestlé Nutrition Institute

Name: Gender:

Age: Weight, kilograms, kg: Height, cm: Date:

Please enter the appropriate scores in the boxes to complete the screening. Sum the screened scores. If the total is 11 points or less, proceed to complete all assessments to determine the "Malnutrition Index Value."

Screening

A In the past three months, has the child reduced food intake due to loss of appetite, digestive issues, difficulty chewing or swallowing?
?
0 = Significant decrease in food intake
1 = Moderate decrease in food intake
2 = No change in food intake

B Weight loss over the past three months
0 = Weight loss greater than 3 kg (6.6 lbs)
1 = Don't know
2 = Weight loss of 1–3 kg (2.2–6.6 lbs)
3 = No weight loss

C
0 = Requires prolonged bed rest or wheelchair use
1 = Able to get out of bed or leave wheelchair, but unable to go outside
2 = Can go outside

D Have you experienced psychological trauma or acute illness in the past three months?
0 = Yes 2 = No

E Psychological Issues
0 = Severe dementia or depression
1 = Mild dementia
2 = No psychiatric/psychological issues

F Body Mass Index (BMI) (kg/m²)
0 = BMI below 19
1 = BMI 19 to less than 21
2 = BMI 21 to below 23
3 = BMI 23 or above

Screening Score (Maximum 14 points)

12–14 points: Normal nutritional status
8–11 points: At risk of malnutrition
0-7 points: Malnutrition

If a more detailed nutritional assessment is needed, please continue to questions G-R.

Assessment

I

more prescription medications daily?
0 = Yes 1 = No

Do you have bedsores or skin ulcers?
0 = Yes 1 = No

J How many main meals do you eat each day?
0 = 1 meal
1 = 2 meals
2 = 3 meals

K Protein Intake Indicator
- Consumes at least one serving of dairy products (milk, cheese, or yogurt) daily Yes Yes
- Eat two or more servings of dried beans or eggs per week Yes No
- Eat meat, fish, or poultry every day Yes No

0.0 = 0 or 1 [Yes]
0.5 = 2 [Yes]
1.0 = 3 [Yes]

L Do you eat two or more servings of fruits or vegetables daily?
0 = No 1 = Yes

M How many fluids (water, juice, coffee, tea, milk...) do you drink daily?
0.0 = Less than 3 cups
0.5 = 3 to 5 cups
1.0 = More than 5 cups

N Eating patterns
0 = Requires assistance to eat
1 = Able to eat independently but with slight difficulty
2 = Able to feed independently

O Self-Assessment of Nutritional Status
0 = Perceived as malnourished
1 = Uncertain about own nutritional status
2 = Perceived no nutritional issues

P How does the patient rate their health compared to others their age?
0.0 = Worse than others
0.5 = Don't know
1.0 = Same as others
2.0 = Better than others

Q Mid-Arm Circumference (MAC) (centimeters, cm)
0.0 = MAC below 21
0.5 = MAC 21 to less than 22
1.0 = MAC 22 or above

R Calf Circumference (CC) (centimeters, cm)
0 = CC below 31
1 = CC 31 or above

Ref.
Vellas B, Villars H, Abellan G, et al. *Overview of MNA® - Its History and Challenges.* J Nut Health Aging 2006; 10: 456-465.
Rubenstein LZ, Harker JO, Salva A, Guigoz Y, Vellas B. Screening for Undernutrition in Geriatric Practice: *Developing the Short-Form Mini Nutritional Assessment (MNA-SF).* J. Geront 2001; 56A: M366-377.
Guigoz Y. The Mini-Nutritional Assessment (MNA®) *Review of the Literature – What does it tell us?* J Nutr Health Aging 2006; 10: 466-487.

® Société des Produits Nestlé SA, Trademark Owners
© Nestlé Products Company SA 1994, Revised 2009.

For more information: www.mna-elderly.com

Value"

Total Assessment Score 24 to 30 points	Normal Nutritional Status
Total Assessment Score 17 to 23.5 points	At Risk for Malnutrition
Total assessment score less than 17 points	Malnutrition

Registered trademark of Société des Produits Nestlé S.A. © Société

MMSE	
Item	Question
Direction	1. What year is it this year? (1 point) 2. What season is it now? (1 point)<br>3. What month is it now? (1 point) 4. What is today's date? (1 point)<br>5. What day of the week is it today? (1 point) 6. Which city are we currently in? (1 point)<br>7. What district does your home reside in? (1 point) 8. Which street (village) do you live on? (1 point)<br>9. What floor are we currently on? (1 point) 10. What place is this? (1 point)
Registration (Word Instant Recall)	11. I will now name three items. After I finish, please repeat them back to me. Remember these three items, as I will ask you again later: "Ball, National Flag, Trees." You can repeat them up to 5 times.<br>Ball (1 point) National Flag (1 point) Trees (1 point)
Mental calculation	12. If you have 100 yuan and spend 7 yuan, how much is left? (After the respondent answers, regardless of whether it's right or wrong) Ask, if you spend another 7 yuan, how much is left? Continue calculating this way until subtracting 5 times. Do not repeat the respondent's answers.<br>93 (1 point) 86 (1 point) 79 (1 point) 72 (1 point) 65 (1 point)<br>(Note: When the patient forgets the number after subtracting 7, do not give prompts like “93 subtract 7”; if the previous answer is wrong but the next answer derived from it is correct, only count one error.)
Word Recall	13. What were the three items I asked you to remember just now? Beach ball (1 point) National flag (1 point) Tree (1 point)
Language Ability	14. What is this? Watch (1 point) What is this? Pen (1 point)<br>15. Please do as written on the next page. (1 point)<br>16. Please say a complete, meaningful sentence. Write down the sentence (1 point)<br>17. Now I will say a sentence, please repeat it clearly: “Forty-four stone lions.” (1 point)<br>18. (The examiner will say the following sentence and give the participant a blank sheet of paper. Do not repeat the instructions or demonstrate): Please hold this piece of paper with your right hand (1 point), then fold the paper in half with both hands (1 point), and then place the paper on your lap (1 point).
Structural imitation	19. Please draw according to the sample. Do not explain the figure (1 point).<br>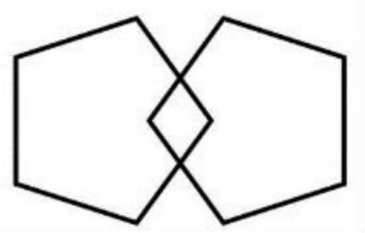

Name ____________________
Sex _______ Age _____
Education _____ Date of exam _______
Administered by ___________________

MONTREAL COGNITIVE ASSESSMENT (MOCA-B) BASIC

	SCORE
**EXECUTIVE FUNCTION**	START TIME ________

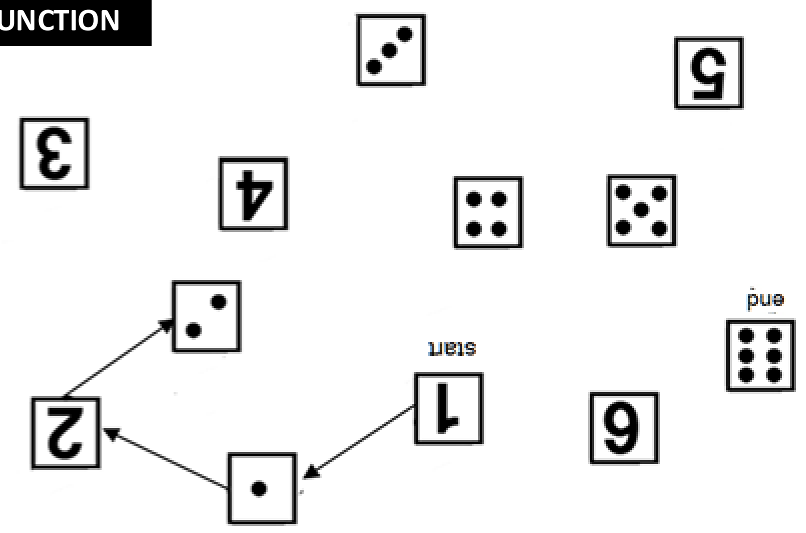

(/1)

IMMEDIATE RECALL

No point

Perform 2 trials even if 1st trial is successful

	ROSE	CHAIR	HAND	BLUE	SPOON
1st trial					
2nd trial					

FLUENCY

Name maximum numbers of **FRUITS** in 1 minute (/2)

1.............	2................	3................	4................	5...............	6...............
7.............	8................	9................	10................	11...............	12...............
13.............	14................	15................	16................	17...............	18...............

N items

2 points if N = 13 or more
1 point if N = 8-12
0 point if N = 7 or less

ORIENTATION

[ ] time (± 2 hr) [ ] day [ ] month [ ] year [ ] place [ ] city (/6)

CALCULATION

Provide 3 ways to pay using 1 dollar coins, 5 dollar and 10 dollar bills for an object that costs exactly **"13 Dollars"** (3 points if 3 ways, 2 points if 2 ways, 1 point if 1 way, 0 point if no correct way) (/3)

[ ] 1................................ [ ] 2. .... [ ] 3. ....

ABSTRACTION

To what category these objects belong to ? (e.g. orange - banana = fruit) (/3)

[ ] train - boat [ ] north - south [ ] drum - flute

DELAYED RECALL

Points are awarded for recall with No cue (1point for each item) (/5)

	ROSE	CHAIR	HAND	BLUE	SPOON
Recall with No cue	[ ]	[ ]	[ ]	[ ]	[ ]
Recall with category cue	[ ]	[ ]	[ ]	[ ]	[ ]
Recall with multiple choice cue	[ ]	[ ]	[ ]	[ ]	[ ]

VISUOPERCEPTION

Identify drawings. No more than 60 seconds. See complementary sheet. (/3)

scissors	T-shirt	banana	lamp	candle
watch	cup	leaf	key	spoon

3 points if N = 9-10
2 points if N = 6-8
1 point if N = 4-5
0 point if N = 0-3
N __

NAMING

Identify animals. See complementary sheet. [ ] zebra [ ] peacock [ ] tiger [ ] butterfly (/4)

ATTENTION

Name the numbers in circles. See complementary sheet. 1 5 8 3 9 2 0 3 9 4 0 2 1 6 8 7 4 6 7 5

ERROR ___N
No point if 2 errors or more (/1)

Name the numbers in circles & squares: 3 8 5 1 3 0 2 9 2 0 4 9 7 8 6 1 5 7 6 4
See complementary sheet. 1 5 8 3 9 2 0 3 9 4 0 2 1 6 8 7 4 6 7 5

ERROR ___ N
2 points if 2 errors or less
1 point if 3 errors
0 point if 4 errors or more (/2)

END TIME ________

TOTAL SCORE (/30)
Add 1 point if education < 4 year AND add 1 point if illiterate
TOTAL TIME ____ min ____ sec

Adapted by : Parunyou Julayanont MD

Copyright : Z. Nasreddine MD

Final Version June 04, 2014

MONTREAL COGNITIVE ASSESSMENT (MOCA-B)

BASIC

COMPLEMENTARY WORKSHEET

VISUOPERCEPTION

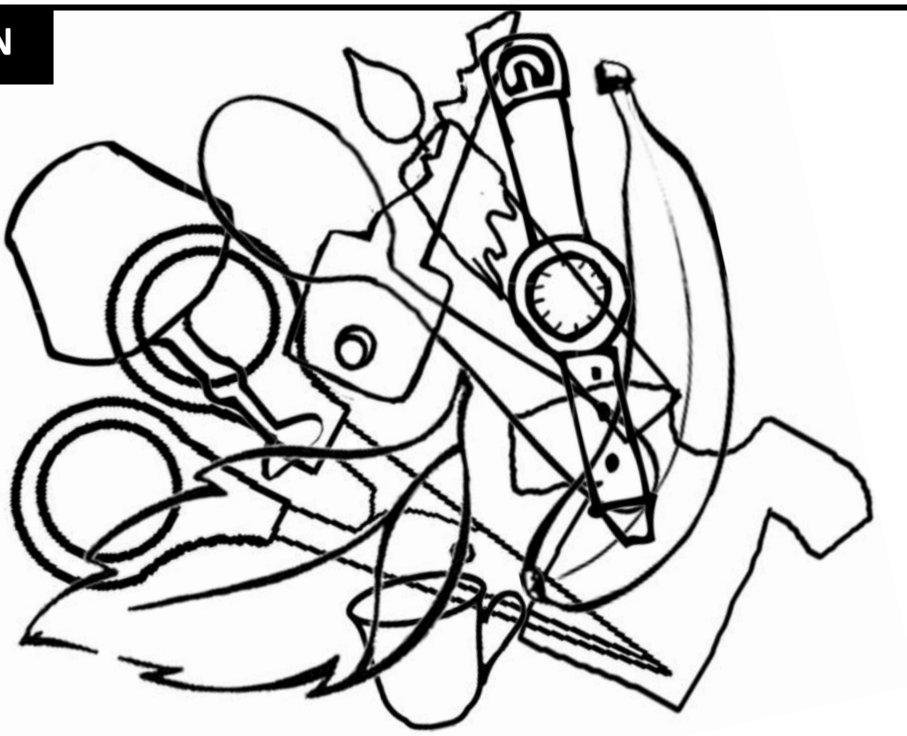

NAMING

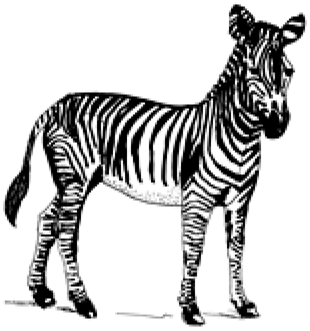

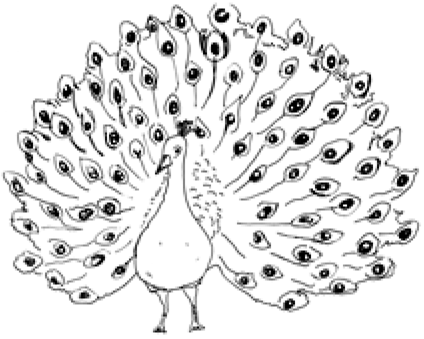

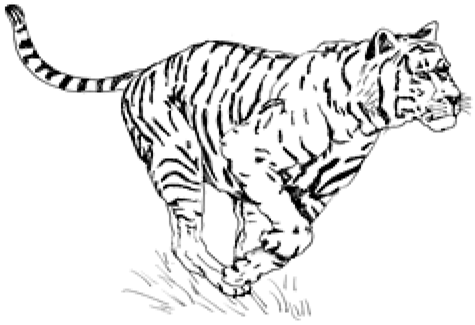

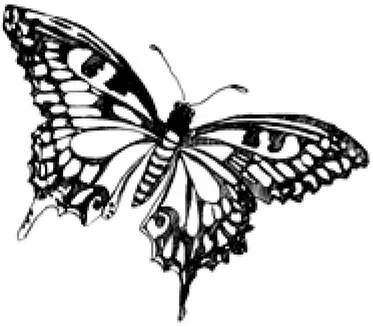

ATTENTION

1 5 8 3 9 2 0 3 9 4 0 2 1 6 8 7 4 6 7 5

3 8 5 1 3 0 2 9 2 0 4 9 7 8 6 1 5 7 6 4

1 5 8 3 9 2 0 3 9 4 0 2 1 6 8 7 4 6 7 5

CLINICAL DEMENTIA RATING (CDR) Patient's Initials_______

CLINICAL DEMENTIA RATING (CDR)	0	0.5	1	2	3

	Impairment				
	None<br>0	Questionable<br>0.5	Mild<br>1	Moderate<br>2	Severe<br>3
Memory	No memory loss or slight inconsistent forgetfulness	Consistent slight forgetfulness; partial recollection of events; "benign" forgetfulness	Moderate memory loss; more marked for recent events; defect interferes with everyday activities	Severe memory loss; only highly learned material retained; new material rapidly lost	Severe memory loss; only fragments remain
Orientation	Fully oriented	Fully oriented except for slight difficulty with time relationships	Moderate difficulty with time relationships; oriented to place of examination; may have geographic disorientation elsewhere	Severe difficulty with time relationships; usually disoriented to time, often to place	Oriented to person only
Judgement & Problem Solving	Solves everyday problems and handles business and financial affairs well; judgement good in relation to past performances	Slight impairment in solving problems, similarities and differences	Moderate difficulty in handling problems, similarities and differences; social judgement usually maintained	Severely impaired in handling problems, similarities and differences; social judgement usually impaired	Unable to make judgements or solve problems
Community Affairs	Independent function at usual level in job, shopping, volunteer and social groups	Slight impairment in these activities	Unable to function independently at these activities although may still be engaged in some; appears normal to casual inspection	No pretence of independent function outside home<br>Appears well enough to be taken to functions outside the family home independent function	Appears too ill to be taken to functions outside the family home
Home & Hobbies	Life at home, hobbies and intellectual interests well maintained	Life at home, hobbies and intellectual interest slightly impaired	Mild but definite impairment of function at home more difficult tasks abandoned; more complicated hobbies and interests abandoned	Only simple tasks preserved; very restricted interests, poorly maintained	No significant function in home
Personal Care	Full capable of self-care		Needs prompting	Requires assistance in dressing, hygiene, keeping of personal effects	Requires much help with personal care; frequent incontinence

Score only as decline from previous usual level due to cognitive loss, not impairment due to other factors.

Reprinted with permission. The Clinical Dementia Rating (CDR) is a copyrighted instrument of the Alzheimer's Diseases Research Center, Washington University, St. Missouri, USA. All rights reserved.
